# Single amino acid change in gp41 region of HIV-1 alters bystander apoptosis and CD4 decline in humanized mice

**DOI:** 10.1186/1743-422X-8-34

**Published:** 2011-01-21

**Authors:** Himanshu Garg, Anjali Joshi, Chunting Ye, Premlata Shankar, N Manjunath

**Affiliations:** 1 Center of Excellence for Infectious Disease, Department of Biomedical Sciences, Texas Tech University Health Sciences Center, 5001 El Paso Drive, El Paso, Texas, 79905 USA; 2 Department of Pediatrics, Texas Tech University Health Sciences Center, 4800 Alberta Ave, El Paso, Texas, 79905 USA

## Abstract

**Background:**

The mechanism by which HIV infection leads to a selective depletion of CD4 cells leading to immunodeficiency remains highly debated. Whether the loss of CD4 cells is a direct consequence of virus infection or bystander apoptosis of uninfected cells is also uncertain.

**Results:**

We have addressed this issue in the humanized mouse model of HIV infection using a HIV variant with a point mutation in the gp41 region of the Env glycoprotein that alters its fusogenic activity. We demonstrate here that a single amino acid change (V38E) altering the cell-to-cell fusion activity of the Env minimizes CD4 loss in humanized mice without altering viral replication. This differential pathogenesis was associated with a lack of bystander apoptosis induction by V38E virus even in the presence of similar levels of infected cells. Interestingly, immune activation was observed with both WT and V38E infection suggesting that the two phenomena are likely not interdependent in the mouse model.

**Conclusions:**

We conclude that Env fusion activity is one of the determinants of HIV pathogenesis and it may be possible to attenuate HIV by targeting gp41.

## Introduction

HIV infection in humans leads to a selective depletion of CD4+ T cells that culminates in immunodeficiency or AIDS. While it is clear that the loss of CD4+ T cells is initiated by HIV infection, the mechanism behind this phenomenon remains highly debated. CD4 T cell loss can occur due to multiple mechanisms: direct killing of infected cells [1], indirect killing of uninfected cells [2], a defect in the capacity for lymphocyte proliferation or turnover or both [3], and/or an overzealous chronic immune response and immune activation [4]. The contribution of these processes to CD4 depletion *in vivo *remains incompletely understood. However, the number of infected cells detectable in HIV-infected individuals is much lower than can account for the profound loss of CD4+ T cells seen with disease progression. Furthermore, SIV infection of the natural hosts in the wild show limited CD4 decline despite active viral replication [5,6], suggesting that virus infection *per se *does not lead to CD4 T cell destruction. Because of these reasons, it has been proposed that apoptosis of uninfected bystander cells may contribute to the depletion of CD4+ T cells [7-9]. In fact, the majority of T cells undergoing apoptosis in peripheral blood and lymph nodes of HIV patients are uninfected [10]. Moreover, massive apoptosis was predominantly observed in uninfected CD4+ T cells present in lymph nodes, thymus or spleen in animal models, such as rhesus macaques infected by SIV or highly pathogenic SIV/HIV chimeric viruses [11,12].

Several HIV-1 proteins, such as HIV envelope glycoprotein Env [13-15], Nef [16,17], Tat [18,19] and Vpr [20,21] can induce T cell apoptosis. However, which of these factors are important *in vivo *is not clear, although cumulative data suggest a major role of Env in cell death of uninfected lymphocytes [22]. Under experimental conditions, Env, either in a soluble [8] or membrane-bound form [23,24], can induce the death of uninfected bystander CD4+ T cells. In Macaque models the membrane fusing activity of the Env glycoprotein has been shown to be critical for CD4 loss [25,26]. However, which mechanism is pertinent for the destruction of CD4 T cells *in vivo *has not been examined under controlled conditions. We have previously characterized a HIV variant with a single amino acid mutation in the gp41 (V38E) that exhibited deficiency in cell-to-cell fusion activity and apoptosis induction *in vitro *as well as increased Enfuvirtide resistance [27]. Using this virus as a model system, in this study, we have compared the pathogenicity of WT and V38E mutant in the humanized mice and find that while both viruses replicate to similar levels and induce immune activation, V38E mutant is compromised in its ability to induce a progressive CD4 T cell loss consequent to its failure to induce bystander apoptosis.

## Methods

### Virus Stock and plasmids

Virus stocks were prepared with molecular clones of the WT virus or V38E mutant as described previously [28]. Briefly, virus stocks were prepared by 293T transfection using infectious molecular clones and Ex Gen 500 transfection reagent. Virus supernatant was collected 48h post transfection, cleared of cellular components by centrifugation, aliquoted and stored at -70°C. WT virus contains the Lai ENV in NL4-3 backbone and the mutant V38E was generated by site directed mutagenesis and have been described previously [27,28]. Virus preparations were quantified using Reverse Transcriptase (RT) activity assay as well as titration in TZMbl indicator cell line (NIH AIDS research and reference reagent program). Enfuvirtide (NIH AIDS research and reference reagent program) resistance was determined in TZMbl cell infection.

### In vitro infection and Apoptosis detection

SupT1 cells (NIH AIDS research and reference reagent program)[29] were infected with equal RT activity units of viruses and cultured for indicated times. The cultures were split 1:3 every other day and culture supernatants were harvested for determination of RT activity. Cells were collected at day 3 or day 5 post infection, fixed and permeabilized and stained with anti-p24 RD-1 antibody clone KC57 (Beckman Coulter) for detection of virus infection and with activated caspase indicator, ZVAD-FITC (Promega) for apoptosis. Flow cytometry was performed on the samples on a FACS CANTO-II flow cytometer. Data was analyzed using FACS DIVA software with at least 20,000 events acquired for each sample. At day 3 or 5 postinfection, cells were also assayed for viability using the Cell Titer Glo (Promega) viability assay. Supernatants from the cultures were assayed for virus replication using RT assay.

### Virus Replication Assay

Peripheral blood was collected from healthy volunteers at Texas Tech University Health Sciences Center (TTUHSC) under protocol approved by the TTUHSC Institutional Review board. PBMCs were separated from whole blood by Ficoll density centrifugation. CD4+ T cells were isolated using negative selection with immunomagnetic beads (Invitrogen). Naïve CD4+ T cells were activated using 5 μg/ml PHA and 25U/ml IL-2 (NIH AIDS Reference and Reagent Program) for 3 days prior to infection. Equal RT units of virus were used to infect the CD4+ PBMCs and cultured for 18 days. Supernatants collected at different time points were assayed for RT activity to determine virus replication.

### Generation of Humanized mice

Humanized BLT mice used in the study were generated as described [30]. Briefly NOD SCID IL2Rγ-/- mice were obtained from Jackson Laboratory (Bar Harbor, ME) and housed at the Texas Tech animal facility as per institutional guidelines. Fetal tissue was obtained from Advanced Bioscience Resources (Alameda, CA). Mice were irradiated with 3gy total body irradiation prior to surgical transplantation of fetal thymus and liver tissue (1-3 mm) under the kidney capsule. CD34+ stem cells were isolated from the fetal liver the same day using positive selection with anti-CD34 coated microbeads (Miltenyi Biotec, Auburn, CA). 5X10^5 ^CD34^+ ^cells were injected in the mice IV following tissue implantation. Human cell expansion and repopulation was determined 10-12 weeks post implantation by multicolor flow cytometric analysis by staining of PBMCs with CD45, CD4 and CD8 antibodies (Beckman Coulter). All use of human tissues and animals was as per institutional guidelines and approved by the Institutional Review Board at TTUHSC.

### Infection of Humanized mice

BLT mice at 12-14 weeks post reconstitution were infected with 50,000 TCID_50 _of virus stocks intraperitoneally. Total of four mice per group were infected with either WT or V38E virus. However one mouse in the V38E and WT group was lost at 2 and 4 weeks respectively during bleeding. Data from all 4 mice is included where available. Peripheral blood was collected from the mice by retro orbital bleeding every 2 weeks for a total of 8 weeks. PBMCs collected at each time point were stained for CD45, CD4, CD8, HLADR, and PD-1. At the 8 weeks end point mice were sacrificed and the spleens were collected and divided in half. One half was fixed in neutral buffered formalin for immunohistopathology. The other half was used for isolation of splenocytes and staining as above. In addition to HLADR and PD-1, staining for CCR5 expression was also performed at the 8 week end point.

### Immunohistopathology

Formalin fixed tissue was paraffin embedded and sectioned. Antigen retrieval was performed using microwave. Sections were stained with FITC conjugated anti- caspase 3 antibody (Beckman Coulter) and RD-1 conjugated anti-p24 antibody KC57 clone (Beckman Coulter) at 1:200 dilution overnight. After extensive washing the slides were stained with DAPI (Antifade) (Invitrogen) and observed under fluorescence microscopy (Nikon Ti Eclipse microscope). Fluorescent images were collected using NIS image acquisition and analysis software (Nikon). Automated quantitation was performed with NIS software to determine total p24, caspase or DAPI stained cells.

### Recovery of virus from PBMCs

DNA was extracted from PBMCs using Qiagen DNA isolation kit. Nested PCR used for amplification of gp41 region has been described by others Aquaro et al [31]. Virus was recovered from PBMCs of infected mice at 8 weeks post infection by coculturing of PHA (5 μg/ml) and IL-2 (25U/ml)-activated PBMCs (5X10^5 ^cells) with 10^6 ^SupT1 cells. RT activity was determined at different time points and supernatants collected for virus sequencing. Formation of syncytia was recorded on day 10 when virus replication was at the peak.

## Results

### V38E virus fails to induce bystander apoptosis in SupT1 cells even in the presence of active viral replication

**Figure 1:**
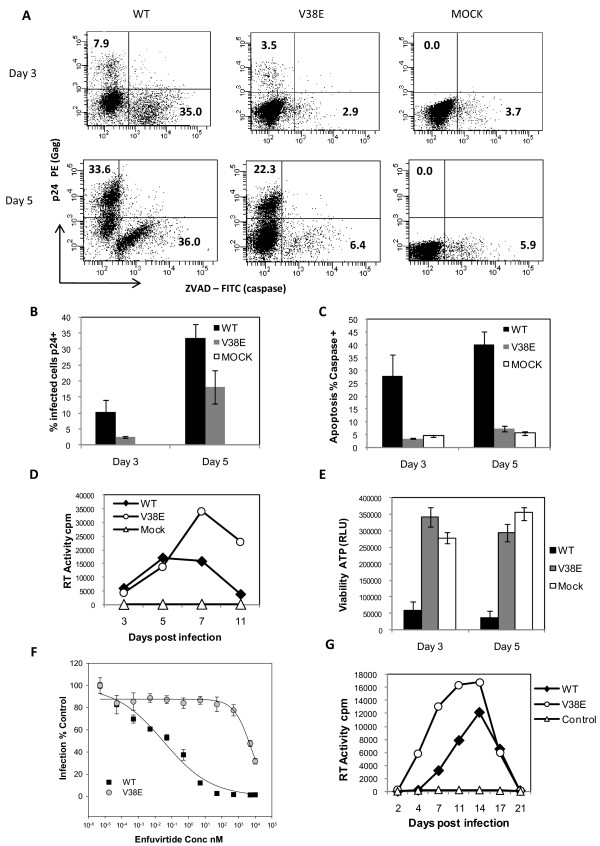
**Bystander apoptosis is induced by WT, but not V38E mutant virus infection in vitro**. (A) SupT1 cells infected with WT or V38E virus were stained with anti-p24 Ab to detect virus infection and ZVAD-FITC to detect apoptosis induction on days 3 and 5 post infection. Cumulative data on virus infection (B) and apoptosis (C) on days 3 and 5 postinfection is shown. (D) Virus replication in the cultures was determined by measuring RT activity in culture supernatants. (E) Cell viability in the cultures was determined by measuring ATP levels in cells using cell titer Glo assay. (F) TZMbl cells were infected with either WT or V38E virus in the presence of indicated concentration of Enfuvirtide. Infection was determined 24h later as luciferase activity and normalized to media control. (G) CD4+ T cells were isolated from whole blood PBMCs and stimulated with PHA (5 μg/ml) and IL-2 (25U/ml) for 3 days. Subsequently the cells were infected with equal RT units of either WT or V38E virus or mock infected and followed for virus replication by determining the RT activity in culture supernatants. All error bars show mean ± SD of triplicate observations.

To test the hypothesis that HIV Env mediated bystander apoptosis and CD4 decline are dependent on gp41 function, we used V38E mutant in comparison to WT virus. V38E Env glycoprotein is restricted in bystander apoptosis induction in coculture experiments where Env expressing cells (Hela-Env) are cocultured with CD4 and CXCR4 expressing target cells (SupT1) [27]. Whether the same would be true in cells infected with HIV remains uncertain. To address this issue, we infected SupT1 cells with either WT or V38E virus and subsequently determined bystander cell death during active virus replication. Cells were stained with p24 (gag) antibody to detect infection and with Z-VAD FITC to detect activated caspase as a marker for apoptosis. As shown in Figure 1A, on days 3 and 5 post infection, numerous p24+ cells were seen in both WT and V38E virus infected cells, confirming infection. However, apoptotic cells were largely restricted to the WT virus infection. Interestingly, a majority of active caspase+ cells in WT virus infected culture were p24 negative, validating that these were in fact bystander cells consistent with data by Holm et al [9]. The fact that under similar experimental conditions V38E mutant failed to induce apoptosis in bystander cells suggests that bystander cell apoptosis induced by HIV infection is dependent on gp41 function. Quantitation of p24 positivity and apoptosis shown in Figure 1B and 1C respectively confirms that while p24+ cells are present in both viral infection, apoptosis is largely restricted to WT virus infection. Although the total percentage of p24+ cells in Figure 1b appears to be higher in WT cultures, this is largely because of loss of cells due to both syncytia formation and apoptosis early on in WT cultures. In fact, viral titers in the culture supernatants, determined by RT activity, was higher for V38E virus than WT on day 7 (Figure 1D). The loss of cells due to syncytia formation and/or apoptosis was also revealed in total cell viability assay by measuring cell-associated ATP (Figure 1E). Interestingly, the loss of viability in WT infected cells was quite significant at both day 3 and day 5, confirming the results seen with the apoptosis marker. Taken together, these findings suggest that V38E mutant is replication competent, yet deficient in inducing bystander apoptosis due to the limited fusion activity of the gp41 glycoprotein. The Enfuvirtide resistance of the V38E mutant was also confirmed in TZM cell line assay (Figure 1F). We next asked whether the replication potential of V38E virus is restricted to cell lines like SupT1 where the receptor and coreceptors are relatively high or the same phenomenon is also true for PBMCs. Infection of activated CD4+ PBMCs with WT or V38E virus showed robust replication by both viruses. Here again we saw that V38E, in fact, replicates much better than the WT virus (Figure 1G), consistent with our hypothesis that gp41 mutants with reduced cell-to-cell fusion activity are unable to induce bystander apoptosis and hence have more targets available for infection. The replication potential of V38E virus in PBMCs also suggested that it would be possible to conduct our studies in humanized mice to test the pathogenesis of the mutant.

### V38E mutant is attenuated in inducing CD4 decline in humanized mice

**Figure 2:**
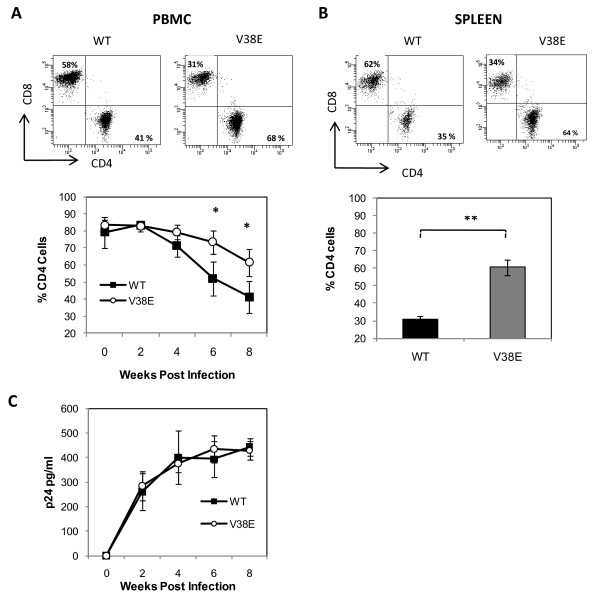
**V38E mutant, compared to WT virus, is limited in inducing CD4 cell decline in the presence of similar levels of virus replication**. Hu-HSC mice were infected with 50,000 TCID50 of viruses and bled every 2 weeks for a total of 8 weeks and then sacrificed. (A) CD4 and CD8 levels in PBMCs from the mice were determined after staining with CD45, CD4 and CD8 antibodies. A representative histogram of PBMC obtained at 8 weeks postinfection (top) and cumulative data on serial CD4 counts (bottom) is shown. (B) Splenocytes collected at 8 weeks postinfection were assayed for CD4 and CD8 levels as above. A representative histogram (top) and cumulative data (bottom) is shown. (C) Plasma collected at the indicated time points postinfection were assayed for vriemia using p24 Ag capture ELISA. n = 3 mice per group. (* p < 0.05. ** p < 0.001)

Various humanized mouse models have been shown to be highly representative for HIV pathogenesis studies [32-34]. Among these models, the BLT mouse model for HIV infection is generated by transplanting human fetal thymus and liver tissue under the kidney capsule of NOD/SCID/IL2Rγ-/- mice followed by iv injection of fetal liver-derived CD34+ hematopoietic stem cells [30]. This model has recently been shown to reflect HIV pathology strikingly similar to humans including high levels of viremia, CD4 decline as well as immune activation associated with virus infection [35,36]. We infected the BLT mice with 50,000 TCID_50 _of either WT or V38E virus and followed them for virus replication and CD4 decline, by measurement of CD4 T cell percentage from before infection to 8 weeks post infection. While peak viremia occurred in both WT and V38E infected mice by 6 weeks, the decline in CD4 counts was significantly higher in WT infected mice compared to V38E virus infected mice (Figure 2A). This difference was also maintained at the 8 weeks end point of our study when we looked at the CD4 levels in the spleen (Figure 2B). In a repeat of the study in a larger set of mice consisting of 6 mice per group the results were identical (Additional file 1, Figure S1), confirming the differential loss of CD4 cells in WT versus V38E infections. However, the circulating viral titers, determined by plasma p24 levels in both the groups was almost identical (Figure 2C), suggesting that the decline was not due to virus infection *per se*, but probably due to differences in the induction of bystander apoptosis. This data also suggests that in the BLT mouse model the decline in CD4 cells is not related directly to virus replication but more so to the phenotype of the Env glycoprotein.

### WT virus infection is characterized by extensive bystander apoptosis in the spleen

**Figure 3:**
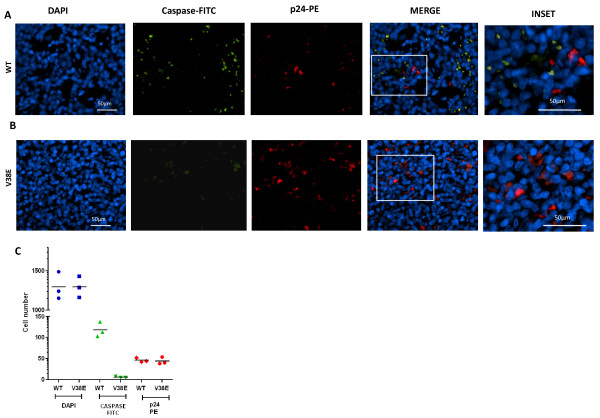
**V38E mutant fails to induce bystander apoptosis *in vivo***. Spleens isolated 8 weeks post infection were fixed in formalin and sectioned. Paraffin embedded sections were stained with anti-p24 RD-1 antibody (red), active caspase 3 antibody (green) and the nuclear stain, DAPI (blue). Individual channels and merge images for WT (A) and V38E mutant (B) infected mice spleens are shown. Enlarged images (right most) from A and B show the presence of apoptotic cells in close proximity to infected cells in WT, but not V38E infected mice. (C) Automated fluorescence quantitation of total (DAPI), apoptotic (Caspase) and infected cells (p24) from at least 6 images from 2 different slides from each mice was performed using NIS elements image analysis software (Nikon). Each symbol represents an individual mouse and the horizontal lines represent the mean.

To directly test whether the differential CD4 decline is due to differences in the induction of bystander apoptosis, we stained spleen sections of infected mice with anti-p24 antibody for detection of infected cells and anti-active caspase-3 antibody as a marker for apoptosis. In the WT virus infected mice, numerous apoptotic cells were detected alongside p24+ cells (Figure 3A). In striking contrast, apoptotic cells were almost undetectable in the spleens of V38E virus infected mice (Figure 3B), even in the presence of similar levels of p24+ cells as in the WT group. Moreover, the apoptotic cells in WT virus infected mice were largely uninfected (p24 negative) bystander cells that were in close proximity to the infected cells (Figure 3A), consistent with the finding in lymph node sections from HIV infected individuals that apoptosis is largely restricted to bystander cells in close proximity to infected cells [10]. Quantitative analysis of at least 6 images from 2 different slides from each mouse confirmed that while both WT and V38E virus showed similar levels of p24 staining consistent with our cell line data and plasma viremia, there was little to no apoptosis in V38E infected mice (Figure 3C), suggesting that a single point mutation in gp41 (V38E) is enough to abrogate bystander apoptosis but not virus replication.

### V38E mutant replicates in humanized mice without reverting to WT

**Figure 4:**
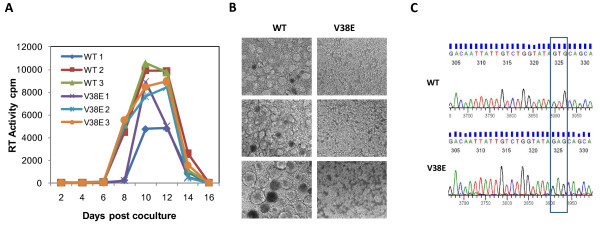
**V38E mutant replicates in Mice without reverting to WT**. (A) Viruses were recovered from mice sacrificed at the 8 weeks post infection by coculturing PHA and IL-2-activated PBMCs with SupT1 cells. Supernatants were harvested at different time points and RT activity determined. (B) Photomicrographs of cultures in (A) on day 10 is shown. Magnification = 10X. (C) Sequence analysis of proviral gp41 from WT and V38E infected mice.

Our hypothesis is that the point mutation in gp41 restricts the Env fusogenic activity and consequently bystander apoptosis and CD4 decline *in vivo*. While the preceding data supports the hypothesis, we wanted to make sure that the V38E mutant had not reverted to WT in the 8-week infection period. To address this issue, we recovered virus from infected mice PBMCs after coculture with SupT1 cells (Figure 4A). At the same time we also isolated DNA from PBMCs and amplified the gp41 region for sequencing [31]. The recovered virus from each of the V38E virus infected mice showed lack of syncytia formation in contrast to WT virus that induced numerous syncytia (Figure 4B). Sequence analysis of proviral DNA also confirmed that the V38E virus had not reverted to WT virus after 8 weeks of infection and that there were no other changes in the gp41 region (Figure 4C). Hence the V38E virus was both genotypically and more importantly phenotypically identical to the input virus. This suggests that the differential pathogenesis of the viruses can be attributed to the point mutation in gp41 and the associated lack of cell-to-cell fusion activity. Taken together, our results suggest that bystander apoptosis in the humanized mouse model significantly contributes to the CD4 decline in HIV infection *in vivo *and that it is most likely dependent on gp41-mediated fusion activity.

### Both WT and V38E viruses mediate immune activation in CD8 cells

**Figure 5:**
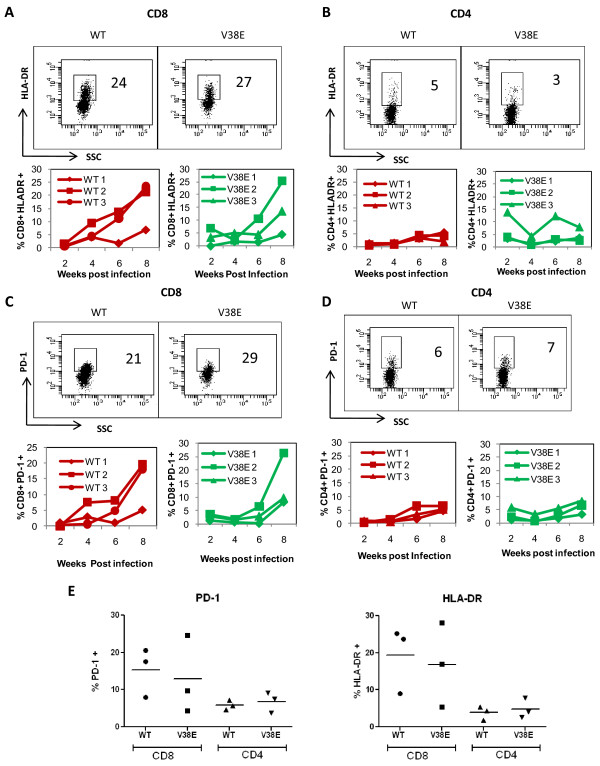
**Immune activation is seen in the CD8+ T cells from both WT and V38E infected mice**. HLADR expression on CD8 (A) and CD4 (B) cells as well as PD1 expression on CD8 (C) and CD4 cells (D) were determined in PBMC obtained from mice at different time points postinfection. A representative histogram at 8 weeks (top) and serial cumulative data from 3 mice (bottom) is shown. (E) Expression of immune activation markers HLADR and PD-1 on splenocytes isolated at 8 weeks postinfection. Each symbol represents an individual mouse.

Immune activation is a hallmark of HIV infection [4] and correlates with CD4 decline in HIV infection [37]. Recently this phenomenon has also been demonstrated in the humanized mouse model [35,36], prompting us to ask whether immune activation correlated with CD4 decline in our study. We looked at HLADR and PD-1, two well established markers associated with HIV disease progression [37,38], on both CD4 and CD8 cells. Interestingly we found that upregulation of HLA-DR (Figure 5A and 5B) and PD-1 (Figure 5C and 5D) was largely restricted to CD8 T cells in both peripheral blood and spleen (Figure 5E) over the 8-week period of our study. The immune activation being restricted to CD8 cells in this model is consistent with other recent studies. More importantly, we found that HLA-DR and PD-1 upregulation was seen in both the WT and V38E infected groups. This suggests that the two viruses were not significantly different in mediating immune activation. The limited CD4 decline in V38E infected group also suggests that immune activation and CD4 decline are probably not interdependent in the humanized mouse model.

### Immune activation correlates with CD4 decline in WT virus but not V38E mutant

**Figure 6:**
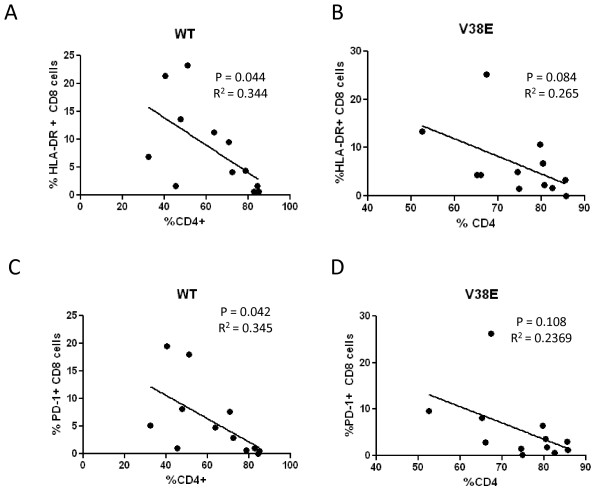
**CD4 decline correlates with CD8 immune activation in WT mice but not V38E mutant**. Correlation of HLADR or PD-1 expression on CD8 cells with CD4 decline in WT and V38E infected groups was determined using Pearson's correlation coefficient. Correlation between HLADR expression on CD8 cells and CD4 decline for WT (A) and V38E (B) is shown. Similarly the correlation between PD-1 expression on CD8 cells and CD4 decline in WT (C) and V38E (D) was also determined.

While the upregulation of immune activation markers is a hallmark of HIV disease we wanted to know whether CD4 decline correlated with immune activation in this model. We conducted a correlation analysis using Pearson's correlation coefficient where we compared CD4 decline to immune activation in CD8 cells. Correlation of CD4 decline and HLADR (Figure 6A and 6B) or PD-1 (Figure 6C and 6D) expression on CD8 cells was determined for both the WT virus as well as V38E mutant. Interestingly we found that the decline of CD4 cells correlates with HLADR (P = 0.044) (Figure 6A) and PD-1 (P = 0.042) (Figure 6C) expression on CD8 cells in the WT group similar to HIV infected patients [37,38] as well as humanized mice [36,39]. However this correlation was not seen in the V38E infected groups (P > 0.05) (Figure 6B and 6D) where CD8 T cell immune activation is seen in the absence of significant CD4 decline. Thus in the BLT mouse model, CD8 T cell activation is most likely mediated by virus replication. Nevertheless the utility of immune activation as a marker for CD4 decline and progression to AIDS under WT infection is validated here.

### CCR5 upregulation in CD8 T cells is similar for WT and V38E infection

**Figure 7:**
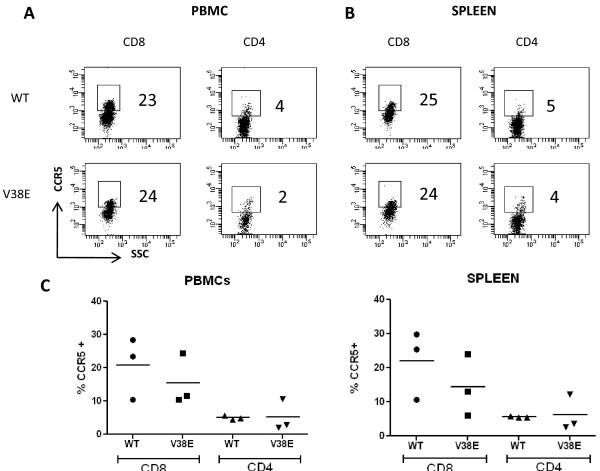
**CCR5 upregulation is seen on CD8 cells in both WT and V38E mutant infected mice**. Mice sacrificed at 8 weeks were assayed for CCR5 expression in both peripheral blood and spleen. Representative histograms of CCR5 expression in CD8 or CD4 cells in PBMCs (A) or Spleen (B) is shown for both WT and V38E Mutant. (C) Cumulative data from 3 mice on the expression of CCR5 on CD4 or CD8 cells at 8 weeks post infection in PBMCs or spleen cells is shown.

CCR5 expression on both CD8 and CD4 cells has also been associated with disease progression in HIV. In the humanized mouse model upregulation of CCR5 on CD8 cells has been reported by others [35,36] although upregulation of CCR5 on CD4 cells is a relatively late event as shown by Brainard et al. We observed that CCR5 was upregulated on CD8 but not CD4 cells at the 8 week end point of our experiment in both PBMCs (Figure 7A) and spleen cells (Figure 7B). Although the CCR5 expression in WT infection was somewhat higher than V38E mutant the results were not statistically significant (Figure 7C). CCR5 expression on CD4 cells on the other hand was relatively low in both infections. These findings suggest that CCR5 upregulation also does not vary between WT and V38E infections although a difference at the later stages of the infection beyond 20 weeks cannot be ruled out.

## Discussion

While the selective loss of CD4 cells over a prolonged period of HIV infection is quiet clear, the mechanism behind this phenomenon remains elusive. We tested the hypothesis that gp41-induced cell-to-cell fusion activity is involved in HIV pathogenesis using an HIV variant with a point mutation in the gp41 region of Env and the humanized mouse model. Compared to WT virus, CD4 loss and bystander apoptosis were both compromised in the V38E mutant. These studies are indicative that the pathogenesis of HIV maybe partially related to the fusogenic potential of the Env glycoprotein. Incidentally, the V38E mutant is one of the Enfuvirtide resistant mutation associated with immunological benefits in patients undergoing Enfuvirtide therapy [31,40]. Whether the lack of bystander apoptosis inducing phenotype in the humanized mice has a relevance to clinical benefits remains to be seen.

Among the several hypotheses proposed for CD4 loss, the role of HIV Env mediated bystander cell death is now gaining strength [22,41]. This is largely due to the fact that Env glycoprotein is expressed on the surface of infected cells, binds to CD4 on bystander cells and can mediate apoptosis [14]. Furthermore, as the depletion of cells in HIV infection is largely restricted to CD4+ T cells and Env binds directly to CD4, a role of Env glycoprotein in further indicated. Although earlier studies suggested that soluble gp120 could induce apoptosis, recent studies point to the importance of membrane expressed Env glycoprotein in bystander apoptosis [41]. The role of gp41 is further strengthened by recent data suggesting that HIV-mediated bystander cell death can be inhibited by gp41 specific fusion inhibitor T20 (Enfuvirtide) [42]. Recent clinical studies have also demonstrated that certain resistant mutants arising in patients undergoing Enfuvirtide therapy are associated with CD4 increase even after virological failure [31,40]. Furthermore, the reduction of fusogenic activity in Enfuvirtide resistant viruses has been demonstrated by Reeves et al [43]. While binding of Env to CD4 as well as a coreceptor CXCR4/CCR5 are both required for apoptosis induction *in vitro*, these interactions alone have been shown to be insufficient for apoptosis induction [44,45]. Using coculture experiments with receptor expressing cells, we and others have recently hypothesized that the fusogenic activity mediated by gp41 is critical for apoptosis induction *in vitro *[28,46-49]. Our *in vitro *data in this study using WT or V38E infected Sup-T1 cells confirms this hypothesis. We find that V38E mutant is incapable of inducing bystander apoptosis in the presence of significant infection and replication in SupT1 cell line. Interestingly bystander apoptosis is seen quiet early in WT infected cultures, suggesting that in fact, a few infected cells can mediate apoptosis of a large number of uninfected bystander cells. Given that the point mutation in gp41 restricts the cell-to-cell fusion capacity of the V38E mutant, while maintaining the virus-cell fusion activity and consequently virus replication, we can state that HIV Env mediated apoptosis is at least in part dependent on Env fusion function. More importantly we can also state that bystander apoptosis is most likely independent of virus replication.

The chronic immune activation and CD4 decline as well as high levels of virus replication seen in the humanized mice makes it a promising model to study HIV pathogenesis [35,36]. However the mechanism of CD4 loss in this model remained unclear and there is no evidence that CD4 loss in this model is associated with apoptosis induction in bystander cells or otherwise. The differential loss of CD4 cells mediated by WT and V38E virus in the presence of similar levels of viremia is a strong indicator of the role of gp41 in CD4 loss. Our study also addressed the question whether bystander apoptosis was involved in the differential CD4 loss between the viruses. Bystander apoptosis was first recognized by Finkel et al in lymph nodes from HIV infected individuals and SIV infected monkeys [10]. Differential bystander apoptosis has also been demonstrated in the nonpathogenic SIVsm infection in Sooty Mangabeys versus SIVmac infection in Rhesus Macaques [6,50]. However none of the previous studies provided any mechanistic insights into this phenomenon. The fact that in our study WT virus infection induces extensive bystander apoptosis that is strikingly absent in V38E virus infection is evidence that the fusogenic activity of the Env glycoprotein may play a key role in bystander apoptosis and consequently CD4 decline *in vivo*.

Another major immunopatholgical change in HIV infection is immune activation [51] that has also been reported in the humanized mouse model [35,36,39]. However the correlation between immune activation and CD4 decline in HIV infection is unclear. Our results show that the immune activation in this model is largely restricted to CD8 T cell over the 8-week period of study similar to recent findings by others [35,36]. Interestingly immune activation between V38E and WT virus infection was similar even though CD4 decline was limited in V38E infection. Correlation analysis revealed that while in WT infection CD8 immune activation correlates with CD4 decline this was not true for V38E infection. Hence, we were able to dissociate immune activation from CD4 decline in this model of HIV infection. Another question is what exactly mediates immune activation in HIV infection. Compromise of the intestinal epithelial barrier and consequent LPS leakage into circulation has been proposed to mediate immune activation in HIV infection [51]. A recent study by Hofer et al further demonstrated that immune activation in the humanized mouse model is a consequence of failure to clear LPS due to a macrophage dysfunction induced by HIV infection [39]. In the same study, experimental induction of immune activation in the absence of HIV infection failed to induce a CD4 decline, suggesting that immune activation may not be the cause of CD4 decline in agreement with our results in this study. It has also been suggested that immune activation in CD8 cells maybe more closely related to active virus replication since the initiation of HAART therapy and reduction in viral load abrogates immune activation in HIV infected individuals [52]. Recently, stimulation of TLR 7/8 by HIV ssRNA has also been proposed as a mechanism of immune activation induced by HIV infection [52,53]. Hence it is not surprising that immune activation was seen with the V38E virus as it replicated to similar levels as WT virus.

Although there are apparent differences between the humanized mice and human infections, and though the findings here cannot be directly extrapolated to HIV pathogenesis in humans, we can still appreciate the differential pathogenesis of a point mutant of HIV. While the use of a laboratory adapted X4 (Lai) isolate is also a limitation of our study the major emphasis of our study is on using the Lai WT and V38E mutant as a model system to study the phenomenon of bystander apoptosis. Our study does provide the first direct evidence of bystander apoptosis as the mechanism of CD4 decline in the humanized mouse model. How relevant are these findings to clinical isolates and HIV pathogenesis in humans remains to be determined. Further studies using viruses directly isolated from patients is likely to address these issues. Furthermore it is known that certain mutations in gp120 that regulate CD4 binding also affect Env fusogenicity [9,54] and in turn could also affect bystander apoptosis *in vivo*. Hence our study supports the idea that targeting different regions of the Env glycoprotein to select less fusogenic variants may have clinical benefits. This study represents a first step in using the humanized mouse model to study differential pathogenesis of HIV-1 Env variants and opens the door to further investigation using this model system.

## Authors' contributions

HG designed the study, performed experiments and wrote the paper. AJ performed the experiments analyzed data and wrote the paper. CY performed experiments. PS and NM designed the study and wrote the paper. All authors read and approved the final manuscript.

## Competing interests

The authors declare that they have no competing interests.

## Supplementary Material

Additional File 1 **Figure S1: Reduced CD4 decline in humanized mice after infection with V38E virus compared to WT**. Humanized mice (6 per group) were infected with either WT or V38E mutant virus. CD4 levels from individual mice (n = 6) for either WT (A) or V38E (B) over a period of 8 weeks was determined. (C) Pooled data from each group shows the striking difference in the CD4 decline between the groups (* p < 0.01, **p < 0.001). Click here for file
