# Supplementary figures and images for: Single amino acid change in gp41 region of HIV-1 alters bystander apoptosis and CD4 decline in humanized mice
Source: Virol J. 2011 Jan 21;8:34. doi: 10.1186/1743-422X-8-34 (PMC3034703; doi:10.1186/1743-422X-8-34)

## Slide 1
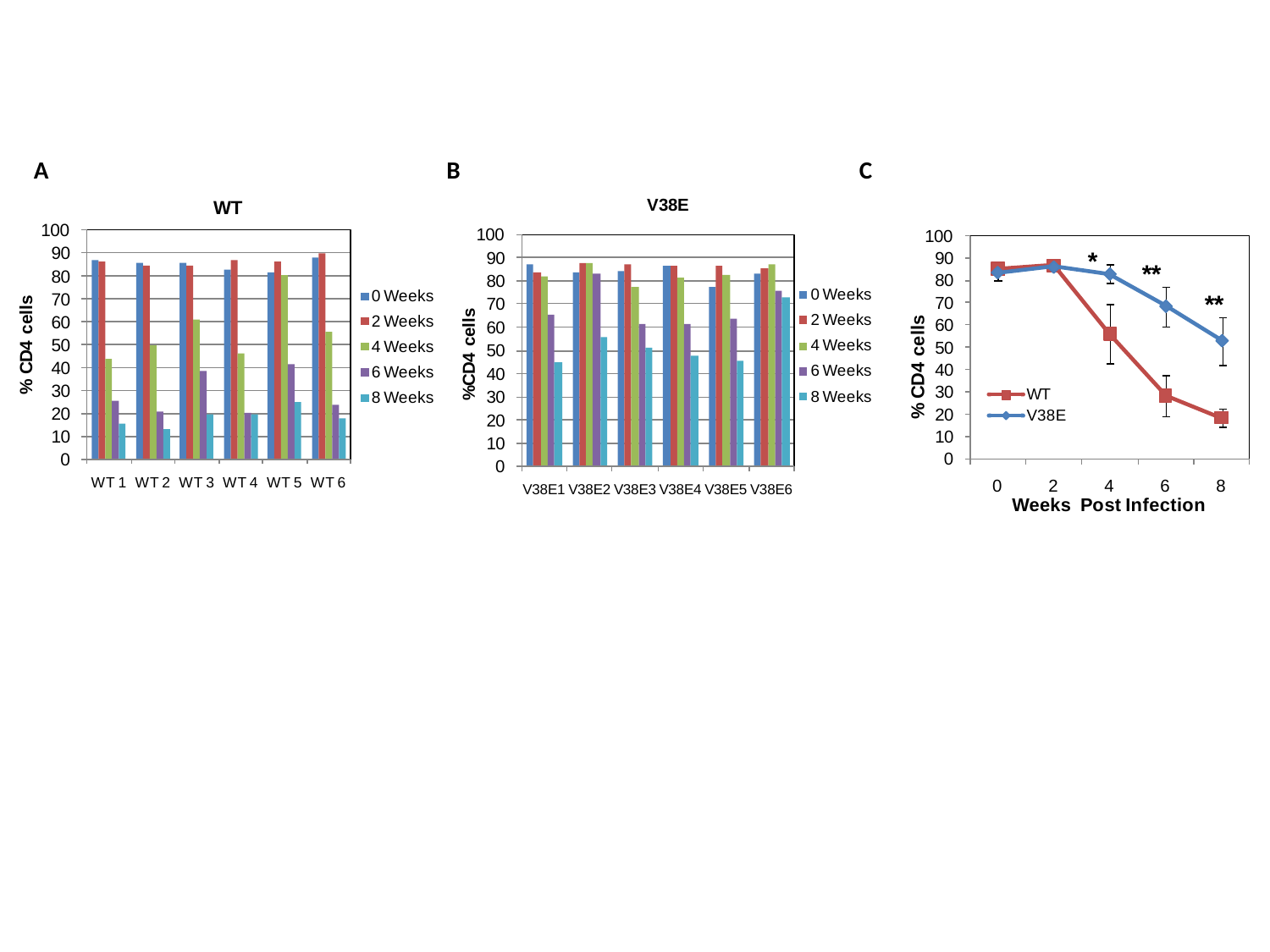

A
B
C

Supplement: Additional File 1 — Figure S1: Reduced CD4 decline in humanized mice after infection with V38E virus compared to WT. Humanized mice (6 per group) were infected with either WT or V38E mutant virus. CD4 levels from individual mice (n = 6) for either WT (A) or V38E (B) over a period of 8 weeks was determined. (C) Pooled data from each group shows the striking difference in the CD4 decline between the groups (* p < 0.01, **p < 0.001). [file 1743-422X-8-34-S1.PPT]
